# PiZ Mouse Liver Accumulates Polyubiquitin Conjugates That Associate with Catalytically Active 26S Proteasomes

**DOI:** 10.1371/journal.pone.0106371

**Published:** 2014-09-11

**Authors:** Christopher J. Haddock, Keith Blomenkamp, Madhav Gautam, Jared James, Joanna Mielcarska, Edward Gogol, Jeffrey Teckman, Dorota Skowyra

**Affiliations:** 1 Edward A. Doisy Department of Biochemistry and Molecular Biology, Saint Louis University School of Medicine, Saint Louis, Missouri, United States of America; 2 Department of Pediatrics, Saint Louis University School of Medicine, Saint Louis, Missouri, United States of America; 3 School of Biological Sciences, University of Missouri – Kansas City, Kansas City, Missouri, United States of America; University of Pittsburgh, United States of America

## Abstract

Accumulation of aggregation-prone human alpha 1 antitrypsin mutant Z (AT-Z) protein in PiZ mouse liver stimulates features of liver injury typical of human alpha 1 antitrypsin type ZZ deficiency, an autosomal recessive genetic disorder. Ubiquitin-mediated proteolysis by the 26S proteasome counteracts AT-Z accumulation and plays other roles that, when inhibited, could exacerbate the injury. However, it is unknown how the conditions of AT-Z mediated liver injury affect the 26S proteasome. To address this question, we developed a rapid extraction strategy that preserves polyubiquitin conjugates in the presence of catalytically active 26S proteasomes and allows their separation from deposits of insoluble AT-Z. Compared to WT, PiZ extracts had about 4-fold more polyubiquitin conjugates with no apparent change in the levels of the 26S and 20S proteasomes, and unassembled subunits. The polyubiquitin conjugates had similar affinities to ubiquitin-binding domain of Psmd4 and co-purified with similar amounts of catalytically active 26S complexes. These data show that polyubiquitin conjugates were accumulating despite normal recruitment to catalytically active 26S proteasomes that were available in excess, and suggest that a defect at the 26S proteasome other than compromised binding to polyubiquitin chain or peptidase activity played a role in the accumulation. In support of this idea, PiZ extracts were characterized by high molecular weight, reduction-sensitive forms of selected subunits, including ATPase subunits that unfold substrates and regulate access to proteolytic core. Older WT mice acquired similar alterations, implying that they result from common aspects of oxidative stress. The changes were most pronounced on unassembled subunits, but some subunits were altered even in the 26S proteasomes co-purified with polyubiquitin conjugates. Thus, AT-Z protein aggregates indirectly impair degradation of polyubiquitinated proteins at the level of the 26S proteasome, possibly by inducing oxidative stress-mediated modifications that compromise substrate delivery to proteolytic core.

## Introduction

Alpha-1 antitrypsin (AT) is the archetype of a large family of serine protease inhibitors (serpins) that use a unique, mousetrap-like, mechanism for inhibiting proteases [1]. The main target of AT is elastase, but AT also inhibits two other neutrophil proteases that degrade the connective tissue of the lungs. This protective function is lost in AT deficiency, an autosomal recessive genetic disorder caused by a mutation in AT gene that results in substitution of lysine for glutamate at position 342 [2]. The mutated protein, called AT-Z, is detected in the serum at only 10–15% of the normal level, which leads to chronic pulmonary disease. In addition, there is a gain-of-toxic-function in the liver, where AT-Z accumulates as highly glycosylated, periodic-acid-Schiff (PAS)-positive and diastase-resistant globules within hepatocytes. This gain-of-toxic-function aspect of AT deficiency is the most common genetic cause of liver failure in children [3]–[6].

Two major proteolytic pathways remove abnormal AT-Z molecules. The molecules that could be still unfolded are removed by the ER-associated degradation (ERAD), a process in which proteins are re-translocated from the ER lumen to cytosolic site of the ER, where they are polyubiquitinated and degraded by the 26S proteasome [7]–[12]. The requirement for unfolding does not apply to autophagy, a lysosomal-like process that removes AT-Z aggregate-filled parts of the ER [13]–[17]. Studies in yeast showed that the contribution of each pathway depends on the expression level of AT-Z, with ERAD being sufficient for cell survival when the expression of AT-Z is low, and with autophagy being necessary when AT-Z expression is high, which would promote aggregation [18]. A key insight into the mechanism of AT-Z aggregation was provided by structural studies [19]–[22], which predicted that E342K substitution would disturb a loop that is normally embedded in AT core and undergoes mousetrap-like rearrangement upon binding to serine proteases. When mutated, this loop would not interact with AT core, instead stimulating formation of dimers, multimers, and polymers. The idea that both soluble and insoluble AT-Z molecules are structured could explain why AT-Z does not induce the classic Unfolded Protein Response that is typically induced by an accumulation of misfolded proteins in the ER [23], [24].

Whereas the role of proteolytic pathways in AT-Z clearance is relatively well understood, less clear is the mechanism of liver injury. One of the best models of the gain-of-toxic-function mechanism of liver damage is the PiZ mouse, in which the human AT-Z gene is expressed from its own promoter in the liver [25]–[27]. Studies of PiZ mouse liver revealed loss of mitochondria due to activated autophagy and signs of damage associated with caspase-3 activation in the remaining mitochondria [28]. The mitochondrial permeability inhibitor cyclosporin A reduced cell death without reducing AT-Z levels, suggesting that the dysfunction of mitochondria is key to the injury [28]. Nevertheless, the damage of mitochondria was enhanced in hepatocytes with high AT-Z content [29] and reduced by pharmacological activators of autophagy that also reduced AT-Z burden [30], [31], showing good correlation with AT-Z levels. AT-Z build-up was also linked to oxidative stress [32], [33] that is common to protein aggregation-related diseases and aging [34]–[37].

Accumulation of protein aggregates has been proposed to facilitate inhibition of the ubiquitin-proteasome system (UPS) [38]–[43]. Since the UPS regulates many aspects of cell biology in addition to misfolded protein clearance [44], it is expected that its inhibition would contribute to pathogenicity. Interestingly, while the UPS is complex and could be inhibited by a variety of mechanisms, many studies concluded that indirect inhibition of the 26S proteasome has to play a major role in this process [45]–[55]. This conclusion is based on evidences such as an impaired degradation of a variety of naturally short-lived proteins and synthetic GFP reporters that were polyubiquitinated, arguing against major defects in ubiquitin-transfer cascades, and an accumulation of polyubiquitin conjugates even in those cellular compartments that were free of the aggregates, implying indirect mechanism. Since polyubiquitination is the main rate-limiting step in ubiquitin-mediated proteolysis, an accumulation of polyubiquitin conjugates typically is interpreted as a defect in proteolysis. However, variable outcomes were obtained in direct measurements of proteolytic rates [56]–[59] and no direct evidence currently links the commonly observed global accumulation of polyubiquitin conjugates to a defect at the 26S proteasome.

In this study, we tested how the conditions of AT-Z related liver injury affects the 26S proteasome using the PiZ mouse, an animal model of the human AT-Z associated liver disease. To address this question, we developed a rapid extraction strategy that preserved polyubiquitin conjugates in the presence of catalytically active 26S proteasomes and allowed their separation from deposits of insoluble AT-Z. Our findings suggest that AT-Z protein aggregates indirectly impair degradation of polyubiquitinated proteins at the level of the 26S proteasome, and that this impairment is associated with oxidative stress-mediated modifications and crosslinking of selected subunits that unfold substrates and regulate access to proteolytic core.

## Materials and Methods

### Ethics statement

The Animal Care and Use Committees at Saint Louis University has approved all animal studies presented in this report. Groups of five mice were housed in micro-isolator cages within the animal facility at the Saint Louis University Doisy Research Center. Mice were maintained on a cycle of 12 hours of light and 12 hours of dark, with lights on at 6:00 am; provided with unlimited water and chow, and remained untreated prior to being euthanized. For isolation of unperfused livers, mice were euthanized using CO_2_ followed by cervical dislocation immediately before liver isolation. For isolation of perfused livers, animals were anesthetized for 5 minutes with pentobarbital sodium (0.26 mg/g of body weight) administered by hepatic portal vein. A total of 19 mice were used in this study.

### Mouse strains, liver isolation, and histological analysis

We used 73–103 days old, male PiZ mice maintained on a C57BL/6 background (NNT+; mouse IDs: PiZ 4603, 4691, 4755, 4797, 4811), and reference C57BL/6 (NNT+; mouse IDs: 1750, 1812, 1819, 1849) or C57BL/6J (NNT-; mouse IDs: 1729, 1798, 1740, AB01, AB02) mice. For biochemical analysis, livers were removed using standard surgical procedures, briefly submerged in PBS, trimmed from ligaments, cut into fragments, and blast frozen in liquid nitrogen. Where indicated, livers were perfused for 10 minutes with a total of 30 ml of warm liver perfusion medium (Invitrogen) prior to liver harvest. Separate liver sections were fixed in formalin and analyzed for diastase-resistant deposits of glycogen using standard histological Periodic Acid Schiff diastase (PASD) protocol [60].

### Antibodies and enzymes

Enzo Life Sciences: purified human 20S (BML-PW8720-0050), rabbit antibodies to dog Calnexin (ADI-SPA-860) and mouse BiP (ADI-SPA-826); mouse antibodies to alpha subunits (BML-PW8195), human Rpt4 (BML-PW8830), human Rpt5 (BML-PW8770), human Rpt6 (BML-PW9265); Abcam: rabbit antibodies to mouse β5 (ab3330); Ambion: mouse antibodies to human GAPDH (AM4300); Sigma: rabbit antibodies to bovine ubiquitin (U5379) and human LC3B (L7543), apyrase (A6535); Cell Signaling Technology: rabbit antibodies to human Cytochrome C (4280); Santa Cruz Biotechnology: rabbit antibodies to human Lamin A/C (sc-20681); Diasorin: goat antibodies to α1 antitrypsin from normal human plasma; Promega: horseradish peroxidase-conjugated donkey anti-rabbit and anti-mouse antibodies; New England Biolabs: Endo H protein (P0702L) and PNGase F protein (P0704S).

### Recombinant Psmd4 protein fragments

Recombinant DNA fragments encoding full length Psmd4 (Psmd4^FL^, base pairs 4–1137, amino acids 2–380); Psmd4 lacking ubiquitin-binding domain (Psmd4ΔUBD, base pairs 4–588, amino acids 2–196); and UBD^only^ (base pairs 619–954, amino acids 197–305) were amplified by PCR (oligonucleotide sequences available upon request) from cDNA MGC-6683 (IMAGE ID: 3581937, ATCC). PCR fragments were subcloned into pUni50, one of the Cre-Lox-based univector plasmid-fusion systems [61], sequenced, and recombined with the Cre-Lox compatible pHI100 expression vector to create in-frame fusion with DNA sequence encoding N-terminal Gst epitope tag. The recombinant Gst fusion proteins were expressed in BL21(DE3) lysS bacteria cells and purified using G^SH^ Sepharose (Sigma-Aldrich) accordingly to the manufacturer's instructions, typically resulting in 45 µg protein per 10 µl beads.

### Small-scale extractions of liver fragments

20 mg fragment of snap-frozen liver was extracted for 1–2 minutes with two tissue volumes (40 µl) of an ice-cold, detergent-free buffer (50 mM Tris, pH 7.2, 50 mM KCl, 5 mM MgCl_2_, 1 mM ATP, and 0.5 mM DTT) followed by centrifugation at 12,000 rpm at 4°C for 1 minute and collection of supernatant/extract. The remaining tissue was extracted in a similar manner four additional times. Since the first three extracts contained the majority of proteins, only the first three extracts were combined, aliquoted, and snap-frozen as “detergent-free” extract that typically had protein concentration of 10 mg/ml for WT and 6 mg/ml for PiZ. The remaining tissue was then extracted using buffer enriched with 0.5% Triton-X-100. Since in this case the majority of proteins were in the first extract, only the first extract was aliquoted and snap-frozen as “extract with Triton”, with protein concentration of 3.5 mg/ml for WT and PiZ. 2% SDS was used to solubilize all proteins in the remaining tissue.

### Western blot

Proteins separated by sodium dodecylsulfate-polyacrylamide gel electrophoresis (SDS-PAGE) were transferred to nitrocellulose by 12-hour electrotransfer at 30 V in Tris/glycine buffer with 5% methanol. Membranes were blocked with fat-free bovine milk and incubated with antibodies diluted in immunoblotting buffer (10 mM Tris, pH 8.0, 150 mM NaCl, and 0.05% Tween 20), for 1 hour or as necessary for quantitative detection established in analysis of serially diluted extracts. Horseradish peroxidase-conjugated secondary antibodies were diluted 1∶25,000, incubated for 25 minutes, washed, and detected by ECL. Reliable detection of polyubiquitin conjugates required that extracts were diluted quickly using ice-cold buffers, that polyacrylamide gels had concentration no higher than 10%, and that electrotransfer was facilitated in containers with plates rather than wires, to ensure the most uniform transfer.

### *In vitro* de-glycosylation by Endo H and PGNase F

De-glycosylation was performed as recommended by the manufacturers, using extracts standardized to contain similar amounts of denatured AT-Z. To prepare denatured extracts with similar amounts of AT-Z, each extract was first adjusted to 2 µg/µl of total protein using the appropriate extraction buffer. The samples were then diluted with denaturing buffer (0.5% SDS, 40 mM DTT) to 0.2 µg/µl of total proteins for detergent-free extracts and extracts with Triton X-100, or 4 ng/µl of total proteins for extract with SDS, followed by boiling for 4 minutes and cooling to room temperature. De-glycosylation was performed at 37°C for 1 hour in 20 µl reaction mixtures that contained 10 µl of the indicated denatured extract standardized by AT-Z content and either 50 units of Endo H in 10 mM sodium citrate pH 5.0, or 50 units of PNGase F in 5 mM sodium phosphate pH 7.5 and 1% NP40.

### Preparation of liver extracts with high protein content for UBD binding and HPLC experiments

200 mg of blast frozen liver fragment was extracted for 2 minutes with 140 µl of ice-cold detergent-free buffer (1∶0.7 tissue to buffer ratio) followed by separation of insoluble components by centrifugation (12,000 rpm, 1 min, 4°C) and snap freezing of the recovered supernatants/extract. This extract typically had 15–30 mg/ml of total proteins and, due to the best recovery of polyubiquitin conjugates, was best for UBD binding experiments. Second extraction of the same tissue resulted in extract with 5–7 mg/ml of total proteins that was used for HPLC.

### HPLC

HPLC was performed at 6°C on Superose 6 10/30 column in HPLC buffer (50 mM Tris, pH 7.2, 50 mM KCl, 5 mM MgCl_2_, 0.2 mM ATP), with a flow rate of 0.250 ml/min and fraction size 500 µl. The individual fractions were collected in tubes pre-filled with 5 µl of 100 mM ATP, to readjust ATP concentration to 1 mM. Separation of 100 µg extract was sufficient for CTL activity tests, but separation of 500 µg extract was necessary for Western blot analysis of each fraction without the need for re-concentration of the collected proteins. This precaution was necessary because re-concentration led to unequal protein recovery from WT and PiZ samples.

### Analysis of chymotrypsin-like (CTL) activity in HPLC fractions

Since storage on ice led to activity loss in WT fractions containing the 26S proteasome (data not shown), fractions were analyzed as soon as collected. 396 µl of the indicated fraction was pre-equilibrated to 37°C for 2 minutes and added to 4 µl of 10 mM Suc-LLVY-AMC in 100% DMSO also pre-equilibrated to 37°C. The reaction mixture of 400 µl was immediately transferred to a semi-quartz cuvette placed in a 37°C-controlled holder of a Varian Eclipse Fluorescence Spectrophotomoter and monitored continuously with excitation at 380 nm and emission of 460 nm for 5 minutes, to ensure that the rate of AMC accumulation was stable. Where indicated, 1 µM epoxomycin was added and the reactions were monitored until the new rate of AMC accumulation has stabilized (typically 5 minutes). The reaction rate per minute was calculated using Varian Eclipse software.

### UBD affinity chromatography

20 µl aliquot of the indicated liver extract with at least 15 mg/ml of proteins was quickly thawed and re-centrifuged at 12,000 rpm for 2 minutes to remove precipitates formed upon thawing. The collected supernatant was incubated on ice for 5 minutes with 4.5 µg of purified ^Gst^UBD protein immobilized on 10 µl of G^SH^-Sepharose beads. Proteins retained on beads were washed 5 times with 1 ml of ice-cold wash buffer (50 mM Tris pH 7.5, 50 mM KCl, 5 mM MgCl_2_, 0.2 mM ATP, 0.5 mM DTT), and analyzed as indicated.

### EM analysis

The 26S proteasomes were co-purified with polyubiquitin conjugates on ^Gst^UBD beads followed by incubation at 37°C for 7 minutes, centrifugation at 12,000 rpm/min for 1 minute and immediate processing of the collected supernatants for EM. Briefly, undiluted 5 µl aliquots were applied to plasma-discharge treated carbon-coated support grids, adsorbed for approximately 30 sec at room temperature, washed with water, and stained with 2% uranyl acetate (unadjusted pH) for approximately 30 sec before blotting and drying. Samples were examined with a JEOL 1200EXII electron microscope, using magnifications of 40,000 or 60,000 to record images on Kodak SO-163 film at either high-dose or minimal-dose conditions. Films displaying several proteasomes per field were scanned using an Epson Perfection V500 Photo flatbed scanner, using resolutions of 846 or 1270 dpi to yield a pixel size of 5Å. All particles recognized as 20S end and side views, and 26S side views, as well as rings and circles of reproducible dimensions, were selected from these scanned images with the EMAN [62] routine Boxer. After eliminating particles that were recognizable but poorly stained, the selected particles were subjected to multiple rounds of reference-free alignment and averaged using SPIDER [63].

## Results

### A major proteasome pool is rapidly extracted in the absence of detergent from snap-frozen liver fragments, and a smaller additional pool is extracted with Triton X-100

To test how the accumulation of human AT-Z mutant protein affects the 26S proteasome in PiZ mouse liver, we first sought to develop a rapid and gentle extraction strategy that would preserve polyubiquitin conjugates in the presence of catalytically active 26S proteasomes.

**Figure 1:**
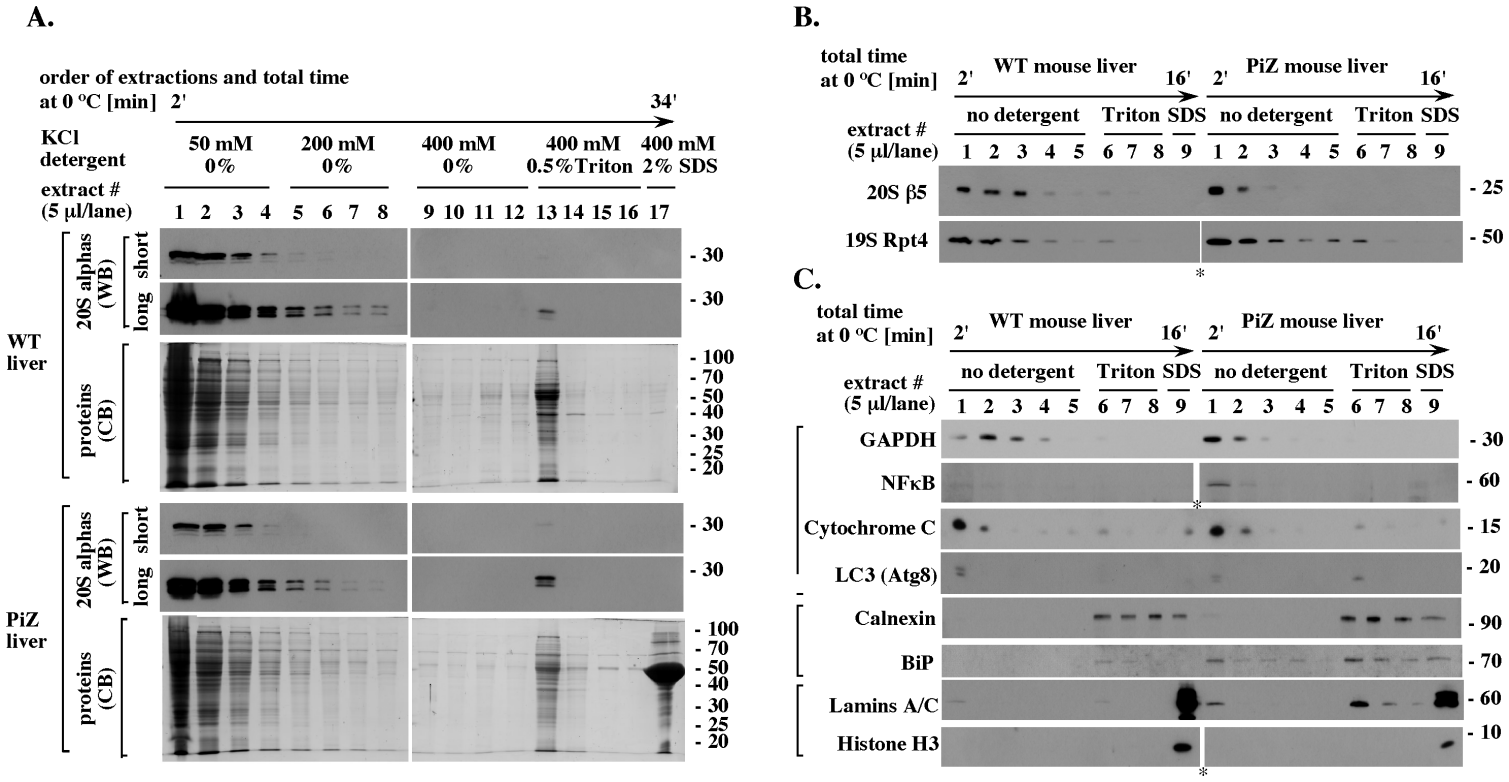
Rapid extraction of the proteasome pools from snap-frozen WT and PiZ mouse liver fragments. (A). Pilot extractions with increasing KCl concentrations in the absence and presence of Triton X-100. A total of 17 extracts, 40 µl each, were prepared sequentially from a single 20 mg WT or PiZ mouse liver fragment, as indicated and described in text. Equal fraction (5 µl) of each extract was separated by 12.5% SDS-PAGE and analyzed by Western blot (WB) for the 20S subunits alpha, or by Commassie blue (CB) for total proteins. Samples shown in lanes 1–8 and 9–17 were analyzed on separate mini-gels that were processed side-by-side and exposed on a single large X-ray film. (B). Extractions with 50 mM KCl in the absence and presence of Triton X-100. Experiment like in A, except that 9 sequential extracts, 40 µl each, were prepared from a single 20 mg liver fragment using buffers that contained 50 mM KCl and either no detergent, 0.5% of Triton X-100, or 2% SDS, as indicated. Asterisks marks a space without sample loaded that was removed from the original 19S Rpt4 WB image to match the loading used in the 20S β5 WB. (C). The origin of the proteasome pools. Experiment like in B, except that with focus on proteins known to reside in specific subcellular locations: GAPDH and NFκB (cytosol); Cytochrome C (mitochondria); LC3 (autophagic vesicles); Calnexin and BiP (ER); Lamins A and C, and Histone H3 (nucleus). The total protein contents in WT and PiZ extracts were verified by Ponceau stain to be similar to those observed in Fig. 1A, CB (data not shown). Asterisk indicates where a lane without sample loaded (empty space) was removed from the original WB image, to match the arrangement of samples in other blots. The data are representative of four WT and four PiZ mice, with 3 randomly selected fragments extracted from each liver (24 extraction sets total).

To establish the extraction strategy, 20 mg fragments of snap-frozen WT and PiZ mouse livers were first incubated on ice for 1 minute with 40 µl of detergent-free hypotonic buffer with 50 mM KCl and 2 mM ATP/Mg^+2^ followed by 1 minute centrifugation at 12,000 rpm and snap-freezing of the recovered extracts. By minimizing the extract preparation time to about 2 minutes and the dilution of cell content to about 2-fold, we expected to prevent possible dissociation of the 26S proteasome into the 19S activator and the 20S proteolytic core. The tissue pellets were extracted four additional times, resulting in four additional extracts prepared sequentially with the same buffer from the same liver fragment. To test whether higher salt concentrations or detergent would elute additional proteasome pools, tissue pellets remaining after the first set of extractions were exposed to similar extractions with 200 mM KCl, 400 mM KCl, and to a combination of 400 mM KCl and 0.5% Triton X-100, each in the presence of ATP/Mg^2+^. The final pellets were boiled with 2% SDS to solubilize all remaining proteins. As a result, seventeen extracts were prepared sequentially from a single liver fragment in about 30 minutes. To determine which of these extracts contained proteasome, we first analyzed an equal fraction of each extract by Western blot. This analysis readily visualized the 20S alpha subunits in detergent-free extracts with 50 mM KCl (Fig. 1A, 20S alphas, WB short, extracts 1–4). Under the same conditions, no major amounts of alpha subunits were detected in extracts prepared with 200 mM KCl and 400 mM KCl (Fig. 1A, 20S alphas, WB short, extracts 5–8 and 9–12). However, longer incubation with antibodies visualized a separate pool of alpha subunits that was extracted by the mixture of 400 mM KCl and 0.5% Triton X-100 (Fig. 1A, 20S alphas WB long, extract 13). The two pools of proteasomal subunits were co-extracted with two well-separated groups of proteins visualized by Commassie blue (Fig. 1A, CB, extracts 1–4 and 13).

Similar results were obtained when buffers had 50 mM KCl and differed in detergent content only. The β5 subunit representative of the 20S proteolytic core and the Rpt4 subunit representative of the 19S activator were detectable mainly in detergent-free extracts (Fig. 1B, extracts 1–3), a small additional pool was extracted by Triton X-100 (Fig. 1B, extract 6), and no additional subunits were extracted by SDS (Fig. 1B, extract 9). To determine the origin of each proteasome pool, extracts were analyzed for proteins known to reside in specific subcellular compartments. Among proteins extracted in the absence of detergent were the cytosolic proteins GAPDH and NFκB, the mitochondrial protein Cytochrome C, and the autophagic protein LC3 (Fig. 1C, extracts 1–4). In contrast, detergent-free extracts did not include the ER proteins Calnexin and BiP, which were extracted by Triton X-100 (Fig. 1C, extracts 6–8), and the nuclear Lamins A and C, or Histone H3, which were extracted by SDS (Fig. 1C, extract 9). Thus, a prominent pool of proteasomes was extracted together with cytosolic, mitochondrial, and lysosomal proteins, and this pool was separated from the proteasomes and other proteins residing in the nucleus and the ER.

### The strategy developed to rapidly extract the proteasome also ensures its separation from deposits of insoluble AT-Z

Compared to antitrypsin (AT), only a small fraction of the total AT-Z mutant produced in the liver is secreted to the serum. The majority of AT-Z is retained in the ER of hepatocytes as a mixture of soluble molecules that can be extracted by Triton X-100 and well-structured polymers that remain insoluble unless exposed to SDS. Thus, the strategy developed to rapidly extract the proteasomes was also expected to separate the proteasomes from deposits of insoluble AT-Z.

**Figure 2:**
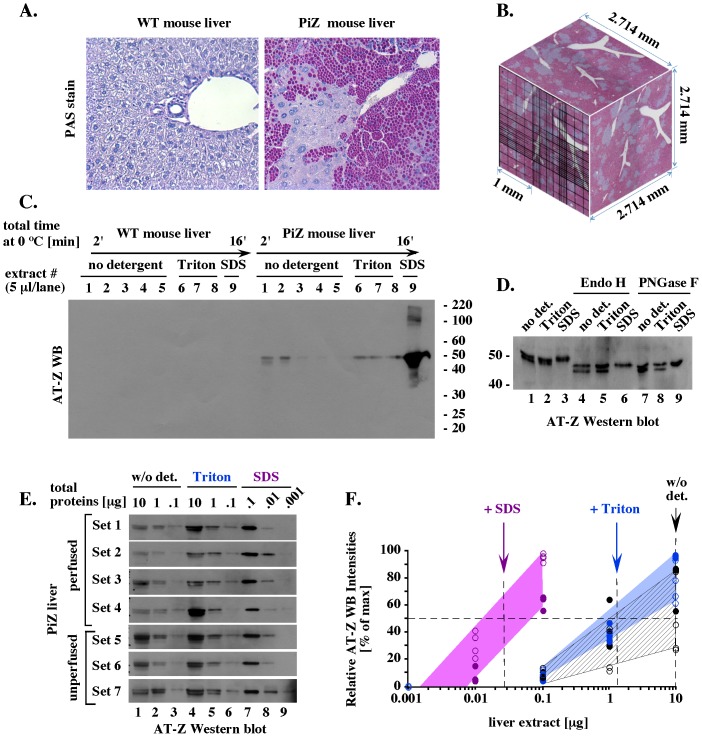
AT-Z analysis. (A). Histological PASD analysis of liver fragments typical of 78 days old WT and PiZ mice. (B). Model of 20 mg liver fragment. The model was prepared assuming liver density similar to water, which in the case of 20 mg fragment would lead to an approximate volume of 20 mm^3^ and a cube root of 2.714 mm. PASD-stained PiZ liver fragments at 50× magnification were fitted into 2.714×2.714 squares with hemacytometer grid. (C). Extraction of AT-Z. Extracts characterized in Fig. 1B and 1C were analyzed by Western blot (WB) for AT-Z. The total protein contents were verified by Ponceau stain to be similar to those observed in Fig. 1A, CB (data not shown). Similar results were obtained with livers isolated from two WT and two PiZ mice, with at least three fragments analyzed from each liver. (D). *In vitro* de-glycosylation of AT-Z. Extracts adjusted to similar amount of AT-Z were treated with the de-glycosylating enzymes as indicated, followed by separation on 8% SDS-PAGE and WB for AT-Z. (E). Western blot analysis of AT-Z levels. The indicated amounts of extracts prepared from seven randomly selected 20 mg fragments of one perfused and one unperfused liver were analyzed by WB for AT-Z. (F). Quantitation of AT-Z Western blots. Data shown in E were quantitated by Image J and presented as a fraction of the highest signal in each set (1–7). Common background highlights symbols related to one type of extract (purple: extract with SDS; blue: extract with Triton X-100; black stripes: extract without detergent; unfilled symbols: perfused liver; filled symbols: unperfused liver). Arrows show the amount of each extract (10, 1.2, and 0.025 µg) required for 50% of the maximal AT-Z signal.

The accumulation of AT-Z in PiZ mouse liver was first verified histologically, using periodic acid-Shiff stain (PAS) with diastase digestion. About 50% of the area in PiZ liver sections had PAS-sensitive globular deposits, while no deposits were found in WT liver (Fig. 2A). This difference in appearance is very similar to the difference observed between normal human liver and human AT-Z liver. The PAS-positive and negative areas were dispersed in the pattern of large clusters that was typical of the whole PiZ liver and well represented in 20 mg liver fragments (Fig. 2B). Thus, extraction of such fragments would average the content of cells with different AT-Z levels, without a bias for any specific content.

Frozen fragments of WT and PiZ mouse livers were then sequentially extracted with buffers containing 50 mM KCl, 2 mM ATP/Mg^+2^, and either no detergent, 0.5% Triton X-100, or 2% SDS. Only a small fraction of AT-Z was extracted from PiZ liver in the absence of detergent (Fig. 2C, extracts 1–2). Incubations with Triton X-100 released another small pool of AT-Z (Fig. 2C, extracts 6–8), but the majority of AT-Z required SDS for solubilization (Fig. 2C, extract 9). The latter pool of AT-Z was also dominating when stained with Commassie blue (Fig. 1A, extract 9, 50 kDa protein). Each pool contained a mixture of AT-Z molecules with diverse molecular weights that were reduced by Endo H and PNGase F treatment (Fig. 2D), verifying that each AT-Z form was glycosylated. The form that required SDS for solubilization had the highest and most uniform weights (Fig. 2D) suggestive of the most heavily glycosylated polymers. The distinct appearance of AT-Z forms that were extracted only upon exposure to SDS is consistent with the interpretation that they represented a unique pool of AT-Z that was insoluble *in vivo*.

To quantitate the amounts of AT-Z, we used an inverse approach aimed at defining the total protein amount of each extract required to detect similar amount of AT-Z. When combined with serial dilutions of each extract, this approach ensured that similar sensitivity of detection was applied to each measurement even when extracts had very different AT-Z content. Similar AT-Z detection was achieved using 10-0.1 µg of extract without detergent or with Triton X-100, and only 0.1-0.001 µg of extract with SDS (Fig. 2E, set 1), suggesting that SDS extract contained about 100-fold more AT-Z than the other two extracts. Additional extracts prepared from three randomly selected 20 mg fragments of the same liver showed similar AT-Z levels (Fig. 2E, sets 1–4). Extracts from unperfused liver had more AT-Z in the detergent-free pool (Fig. 2E, lanes 1–3 in sets 1–4 and 5–7), suggesting that this pool included AT-Z from the serum.

Quantitation of AT-Z levels was also performed by Image J and presented as a fraction of the highest signal in each set of extracts (Fig. 2F). From this graph we estimated that, on average, 50% of the highest signal was detected in 0.025 µg of extract with SDS, 1.2 µg of extract with Triton X-100, and 10 µg of detergent-free extract (Fig. 2F, arrows). Compared to detergent-free extract, extract with Triton X-100 had about 8-fold more (10∶1.2) and extract with SDS had about 400-fold more (10∶0.025) AT-Z. The ratio of 1∶8∶400, equivalent to 0.2∶2∶97.8%, showed that the vast majority of AT-Z (99.8%) required a detergent for solubilization. Thus, insoluble AT-Z molecules were not extracted under conditions that allowed isolation of a major proteasome pool.

### The rapid extraction strategy preserves polyubiquitin conjugates and allows their quantitation

Ubiquitin-mediated proteolysis is a relatively slow process, with protein half-life in the range of minutes to hours. Thus, a major advantage of rapid extraction in the absence of protease inhibitors and denaturing agents would be the isolation of polyubiquitin conjugates that could be further analyzed in functional *in vitro* tests. To address this possibility, we first analyzed the WT and PiZ mouse liver extracts for the presence of high molecular weight polyubiquitin conjugates.

**Figure 3:**
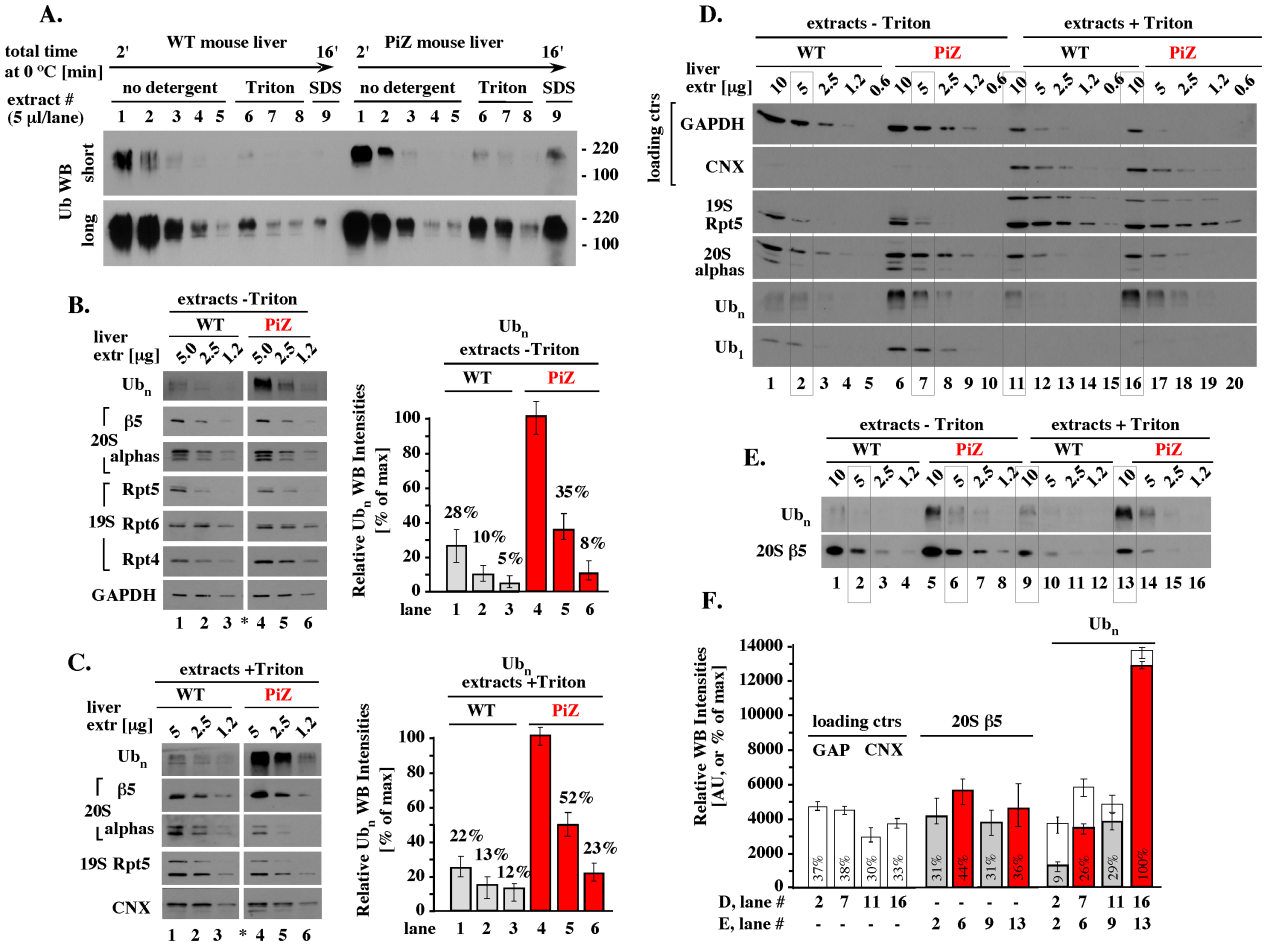
Polyubiquitin pools and load per proteasome. (A). Polyubiquitin pools. 9 sequential extracts were prepared from each 20 mg liver fragment as described in Methods, and an equal fraction of each extract (5 µl our of 40 µl) was analyzed by WBs with antibodies specific to ubiquitin. The total protein contents in WT and PiZ extracts were verified by Ponceau stain to be similar to those shown in Fig. 1A, CB (not shown). (B). Quantitative analysis of polyubiquitin conjugates and proteasomal subunits in detergent-free extracts (extracts - Triton). Left: Extracts 1-3 identified in A to contain the majority of polyubiquitin conjugates were combined to represent ‘detergent-free extract’, as described in Methods. Left: the indicated amounts of detergent-free WT and PiZ extracts (5, 2.5, and 1.2 µg of total proteins) were analyzed by WB on the same membrane, with multiple membranes used to probe for all proteins shown. Asterisk indicates where a lane was removed from the original image, to match the arrangement of samples in other blots. Right: Ub_n_ Western blot intensities were quantitated by Image J and presented as % of the highest intensity. (C). Quantitative analysis of polyubiquitin conjugates and proteasomal subunits in extracts with Triton (extracts + Triton). Experiment like in B except that extract 6 from each set shown in A contained the majority of polyubiquitin conjugates and was used as ‘extract with Triton X-100’ without combining with extracts 7–8, which would dilute this pool (see Methods), and the sensitivity of Western blots was increased by using higher concentration of primary antibodies to ensure detection of lower proteasome levels per similar total protein content. (D). Analysis of polyubiquitin burden per 20S core. The indicated amounts of total proteins in each extract were analyzed side by side on a single membrane to identify the amounts of extracts with similar 20S levels and to estimate the polyubiquitin load per 20S (see text for details). Gray boxes mark samples similar to those marked with gray boxes in panel E. Longer exposure was required for detection of Ub_1_ than Ub_n_. (E). Analysis of polyubiquitin burden per 20S core. Experiment like in D except that samples were probed for polyubiquitin (Ub_n_) and the 20S β5 subunit. (F). Quantitation of polyubiquitin burden per 20S core. The indicated WB data from panels D (white bars) and E (colored bars) were quantitated and shown in arbitrary units (AU) and as a percentage of the highest intensity (12,865 AU = 100%). Note that due to vastly different polyubiquitin levels, analysis under less quantitative conditions (Ub_n_, white bars, longer WB exposure) underestimates differences observed under strictly quantitative conditions (Ub_n_, colored bars, short WB exposure). GAPDH (or GAP), and Calnexin (CNX): loading controls. Error bars represent variations observed in similar analysis performed with livers isolated from three WT and three PiZ mice.

The majority of WT polyubiquitin conjugates were extracted in the absence of detergent, with lower amounts extracted by Triton X-100, or SDS (Fig. 3A, WT extracts 1–5, 6–8, and 9). Similar pools were found in PiZ extracts (Fig. 3A, PiZ extracts 1–5, 6–8 and 9). Two approaches were then used to quantitate the polyubiquitin pools. In the first approach, we analyzed 2-fold dilutions of each extract standardized by total protein content. In detergent-free extracts, detection of polyubiquitin conjugates was linear in analysis of 5 and 2.5 µg of PiZ and WT extracts (Fig. 3B, Ubn WB and graph). The amount of polyubiquitin conjugates was on average 3.6 (+/−0.5)-fold higher in PiZ than WT samples, with 3.6 and 3.5 differences found in analysis of 5 and 2.5 µg proteins, respectively (Fig. 3B, Ub_n_ WB and graph). For extracts prepared with Triton X-100, detection of polyubiquitin conjugates was also linear in analysis of 5 and 2.5 µg proteins, but the sensitivity of Western blots had to be increased by using higher concentrations of antibodies due to the lower proteasome levels per similar protein content. Under those conditions, about 4.2 (+/−0.5)-fold higher amount of polyubiquitin was observed in PiZ than WT extracts, with 4.5 and 4.0 differences found in analysis of 5 and 2.5 µg proteins, respectively (Fig. 3C, Ub_n_ WB and graph). In each experiment, WT and PiZ samples had similar levels of loading controls (GAPDH or Calnexin) and proteasomal subunits, including the 20S subunits β5 and alpha, and the 19S subunits Rpt4, Rpt5 and Rpt6 (Fig. 3B and C).

In the second approach, we sought to estimate the burden of polyubiquitin conjugates per 20S core in each proteasome pool, the major pool extracted without detergent and the minor pool extracted with Triton X-100. To identify the amount of each extract with similar proteasome input, we analyzed two-fold serial dilutions of each extract standardized by total protein content, using GAPDH to verify the loading of extracts without detergent, and Calnexin (CNX) to verify the loading of extracts with Triton X-100 (Fig. 3D, loading ctrs). We focused on the 20S core rather than the 19S activator, as the 19S subunits appeared to be altered in extracts prepared with Triton X-100 (Fig. 3D, 19S Rpt5). The basis of these changes was unknown, but they verified that the extracts contained distinct proteasome pools. On average, most similar levels of the 20S core were present in 5 µg of detergent-free extracts and 10 µg of extracts prepared with Triton X-100 (Fig. 3D, 20S alphas, lanes 2, 7, 11, 16; and Fig. 3E, 20S β5, lanes 2, 6, 9, 13). In the case of the 20S β5 subunit, these amounts of extracts contained about 31, 44, 31 and 36% of the maximal Western blot intensity, respectively (Fig. 3F, 20S β5). The same samples had very different levels of polyubiquitin (Fig. 3E, Ub_n_, lanes 2, 6, 9, and 13), accounting for 9, 26, 29 and 100% of the highest intensity, respectively (Fig. 3F, Ub_n_). Thus, each PiZ extract had on average 3.2 (+/−0.3)-fold more polyubiquitin compared to its WT counterpart (26% versus 9%, and 100% versus 29%). In addition, about 2-fold increase in the levels of monomeric ubiquitin was detected in detergent-free PiZ extracts (Fig. 3D, Ub_1_). The increased load of polyubiquitin in extracts with Triton X-100, which included ER proteins, would be consistent with the location of AT-Z proteolysis. However, the increased load of ubiquitin and polyubiquitin in detergent-free extracts, which contained the majority of total proteasomes, suggested a widespread strain on the ubiquitin-proteasome system in PiZ mouse liver.

### Polyubiquitin conjugates from WT and PiZ livers have similar affinities to ubiquitin-binding domain and associate with similar amounts of catalytically active 26S proteasomes

**Figure 4:**
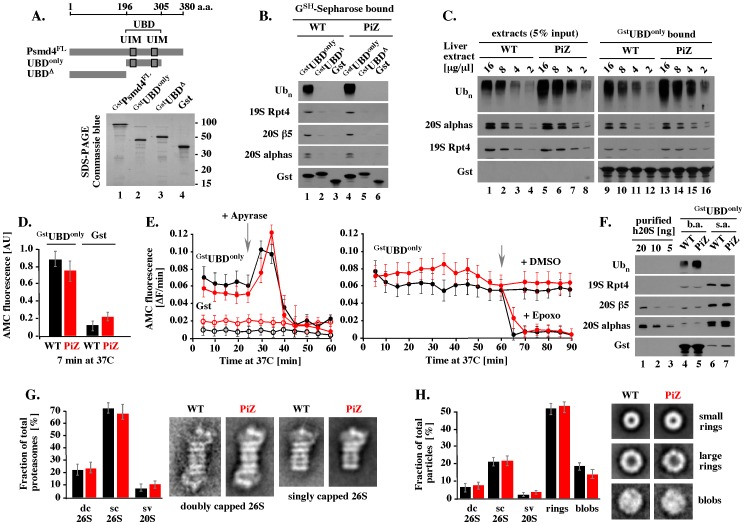
Polyubiquitin conjugates extracted from WT and PiZ livers have similar affinities to ubiquitin-binding domain and associate with similar amounts of catalytically active 26S proteasomes. (A). Psmd4 fragments used in this study. Top: Gst was fused to C-terminal ends of the indicated fragments of mouse Psmd4, as described in methods. FL: full length; UDB: ubiquitin-binding domain; UIM: ubiquitin-interacting motif; Δ: deletion; aa: amino acid. Bottom: proteins retained on 10 µl of G^SH^-Sepharose beads were released by boiling with 20 µl of Laemmli buffer followed by separation of 2 µl by SDS-PAGE and staining with Commassie blue. (B). Binding of polyubiquitin conjugates from detergent-free WT and PiZ extracts. The indicated Gst fusion proteins immobilized on 10 µl of G^SH^ Sepharose beads were incubated on ice with 20 µl of undiluted (16 µg/µl) WT or PiZ liver extracts. After 5 minutes, beads were washed 5 times with 1 ml of ice-cold binding buffer, released by boiling with 10 µl of Laemmli buffer, and analyzed by Western blot, as indicated. (C). Binding of polyubiquitin conjugates from serially diluted WT and PiZ extracts. Binding was performed as described in B, except that ^Gst^UBD^only^ beads were incubated with 20 µl of 2-fold serial dilutions of each extract standardized by total protein content, as indicated. Data shown in lanes 1–8 and 9–16 were derived from separate membranes that were processed and developed side by side. (D). CTL peptidase activity associated with proteins co-purified with polyubiquitin conjugates on ^Gst^UBD^only^ beads. WT and PiZ liver proteins were isolated on 10 µl of ^Gst^UBD^only^, or the control Gst beads, as described in B, followed by suspension in 400 µl reaction mixtures with Suc-LLVY-AMC and incubation at 37°C with gentle mixing. After 7 minutes, beads were sedimented by 10 second spin at 12,000 rpm and supernatants were analyzed for AMC fluorescence, as described in Methods. Error bars show a typical range of variations observed in multiple experiments. (E). Continuous measurements of AMC fluorescence in reaction mixtures separated from beads after the initial 7 minutes of incubation at 37°C. Red symbols: PiZ samples prepared using ^Gst^UBD^only^ (filled) or Gst (open) bites. Black symbols: WT samples prepared with ^Gst^UBD^only^ (filled) or Gst (open) bites. The data are representative of 2–3 independent experiments, and error bars indicate a typical range of variations. (F). Western blot analysis of proteins present in reaction mixtures analyzed in E. Proteins recovered from beads (beads after: b.a.) and supernatants (supernatants after, s.a.) separated after the initial 7 minutes of incubation at 37°C were analyzed by Western blot, as indicated. The indicated amounts of purified human 20S (h20S) are shown as reference. (G). EM analysis of the 26S and 20S side views. Samples were prepared like in E, but processed for EM analysis, as described in Methods. Average images of the doubly capped (dc) and singly capped (sc) 26S structures, and the side view (sv) of the 20S include 83–367 individual particles found in multiple samples. Bars show quantitative representation of each type of structure calculated as a percentage of the total number of the proteasomal side views (584 in WT samples, and 370 in PiZ samples). (H). EM analysis of additional particles in WT and PiZ samples. Average images of small rings, large rings, and blob structures include 15–200 particles found in each sample. Bars show quantitative representation of each type of structure, including the side views of the proteasome, calculated as a percentage of the total number of particles (1192 in WT samples, and 1139 in PiZ samples). Error bars represent variations observed in analysis of three independently prepared sets of samples.

To get an insight into the mechanism by which polyubiquitin conjugates were accumulating in PiZ mouse liver, we first tested their interaction with the ubiquitin-binding domain (UBD) of Psmd4, also called S5a, Mcb1, or Rpn10, the classic polyubiquitin-binding receptor of the 26S proteasome. Full-length Psmd4 (Psmd4^FL^), ubiquitin-binding domain of Psmd4 only (UBD^only^), and Psmd4 fragment that lacks UBD (UBD^Δ^) were fused to Gst, expressed in bacteria, and purified on G^SH^-Sepharose (Fig. 4A). A brief, 5 minute incubation on ice with WT and PiZ extracts followed by quick wash with ice-cold buffer was sufficient to isolate polyubiquitin conjugates on ^Gst^Psmd4^FL^ (data not shown) and ^Gst^UBD^only^, but not on control ^Gst^UBD^Δ^ or Gst beads (Fig. 4B, Ub_n_). In binding assays with 2-fold serial dilutions of each extract standardized by total protein content, similar amounts of polyubiquitin conjugates were isolated with similar polyubiquitin inputs (Fig. 4C, Ub_n_, compare inputs in lanes 2, 3 and 7, 8; ^Gst^UBD^only^-bound fractions in lanes 10, 11 and 15, 16). Thus, polyubiquitin conjugates extracted from WT and PiZ mouse livers had similar affinities to recombinant ubiquitin-binding domain of Psmd4.

In each UBD binding experiment, polyubiquitin conjugates co-purified with endogenous 26S proteasome, as suggested by Western blot analysis with antibodies specific to the 19S Rpt4 subunit and the 20S subunits β5 and alpha (Fig. 4B, lanes 1 and 4; and Fig. 4C, lanes 9–12 and 13–16). To determine whether these 26S proteasomes were active, mouse liver proteins retained on 10 µl of ^Gst^UBD^only^ were suspended in 400 µl reaction mixtures with Suc-LLVY-AMC reporter that emits AMC fluorescence after cleavage by the chymotrypsin-like (CTL) active site, and similar reactions were prepared with mouse liver proteins retained on control Gst beads. After 7 minutes of gentle mixing at 37°C, the beads were sedimented by a brief centrifugation and the amount of AMC accumulated in solution was measured in a fluorescence spectrophotometer. Within experimental error, similar AMC fluorescence was observed in reactions with WT and PiZ proteins retained on ^Gst^UBD^only^ beads (Fig. 4D, ^Gst^UBD^only^), and 5–8 fold less AMC fluorescence was observed in control reactions with WT and PiZ proteins retained on Gst beads (Fig. 4D, Gst).

The beads-free solutions used in the end-point peptidase activity measurements above continued to accumulate AMC fluorescence, thereby allowing real time analysis of catalytic rates. These rates were sensitive to apyrase, an enzyme that depletes ATP (Fig. 4E, left), and 1 µM epoxomycin, one of the most specific inhibitors of the proteasome (Fig. 4E, right) [64], [65]. To verify that the 26S proteasomes were released from ^Gst^UBD^only^ beads after the initial 7 minutes of incubation at 37°C, we compared proteins recovered from AMC measurements with proteins retained on ^Gst^UBD^only^ beads. The ^Gst^UBD^only^ beads retained polyubiquitin conjugates, but most of the 26S proteasomes were released (Fig. 4F, beads after, b.a., and supernatants after, s.a., lanes 4–7). By comparison to known amounts of human 20S, the activity assays contained about 20 ng of β5 subunit (Fig. 4F, lanes 6, 7, and 1), which would be equivalent to about 0.5 pmol of the 20S. Assuming that all 20S cores were involved in ATP-dependent reactions, 28 pmol AMC would be liberated per minute per pmol of the 26S proteasome in WT and PiZ samples.

Analysis by electron microscopy also did not reveal major differences between WT and PiZ proteasomes released from ^Gst^UBD beads. Visual classification of particles (3131 total) in multiple experiments revealed similar distribution of the most easily identifiable side views, with 21.7% and 22.7% of doubly capped 26S, 71.3% and 67.0% of singly capped 26S, and 7.0% and 10.5% of uncapped 20S in WT and PiZ samples, respectively (Fig. 4G). In this classification, 93%–90% of the total proteasomes initially co-purified with polyubiquitin conjugates contained at least one 19S cap. In addition, we observed amorphous ‘blobs’, which were similarly abundant in each sample (Fig. 4H, blobs), and two types of rings that, together, accounted for about 52.0% and 54% of the total WT and PiZ particles, respectively (Fig. 4H, rings). One type of ring had a diameter similar of the end view of the 20S core (Fig. 4H, small rings). Another type of ring was larger and displayed 8-fold symmetry inconsistent with the seven-fold symmetry of the 20S end view (Fig. 4H, large rings). These larger rings may represent the p97/VCP/Cdc48 ATPase that binds polyubiquitin chains and has multiple roles, including a role in ubiquitin-mediated proteolysis [66]. Overall, not only the 26S proteasomes, but also other large particles that co-purified with polyubiquitin conjugates appeared to be similar in WT and PiZ mouse liver samples.

### WT and PiZ mouse liver extracts have similar, albeit not identical, pools of the 26S, 20S, 19S, and unassembled subunits

Since the 26S proteasomes co-purified with polyubiquitin conjugates were likely to represent only a fraction of the total proteasomes, we analyzed all proteasome pools by size exclusion chromatography. The analysis was performed on Superose 6 HPLC column pre-equilibrated at 6°C with a standard, detergent-free, extraction buffer including ATP/Mg^+2^.

**Figure 5:**
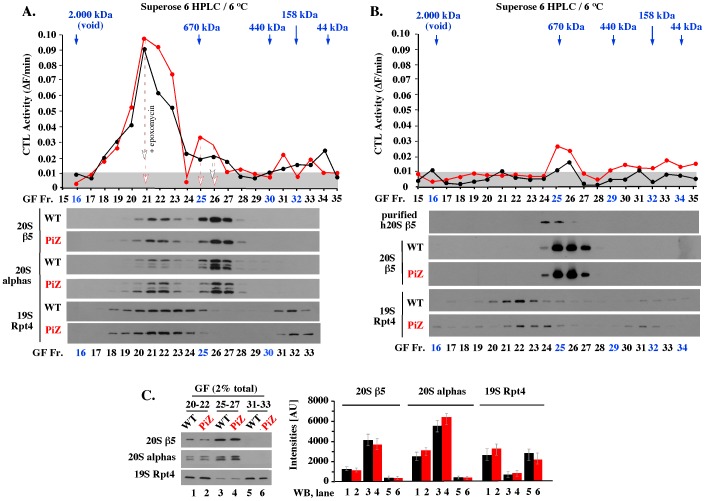
Gel filtration chromatography reveals similar, albeit not identical, pools of the 26S, 20S, 19S, and unassembled subunits in WT and PiZ mouse liver extracts. (A). HPLC of freshly thawed, detergent-free WT and PiZ liver extracts. WT (black) and PiZ (red) extracts were separated by HPLC on Superose 6 10/30 and analyzed for CTL peptidase activity (top, 100 µg of total protein input) or Western blot (bottom, 500 µg of total protein input) as described in Methods. Arrows mark the loss of activity after incubation with 1 µM epoxomycin, or apyrase that depletes ATP. (B). HPLC of WT and PiZ liver extracts pre-incubated for 1 hour at 37°C with 200 mM KCl. Experiment like in A, except that extracts were pre-incubated for 1 hour at 37°C with 200 mM KCl, to induce separation of the 20S and 19S complexes. (C). Quantitation of the indicated 20S and 19S subunits in the three major pools separated by HPLC (GF 20–22, GF 25–27, and GF 31–33). The indicated fractions (GF 20–22, GF 25–27, and GF 31–33) were combined and analyzed side-by-side by Western blot, as indicated. Quantitation of WB data is shown on the right, with error bars indicating the range of variations in 3 experiments.

CTL peptidase assays with the Suc-LLVY-AMC reporter revealed similar, albeit not identical, activity profiles in WT and PiZ fractions separated on Superose 6. In each case, the most prominent peak of activity eluted at the apparent molecular weight of the 26S proteasome, which ranges from about 1,200 kDa to 1,800 kDa, depending on whether the 20S complex is associated with one or two 19S activators (Fig. 5A, GF 19–23). This proteolytic activity was sensitive to epoxomycin, an inhibitor specific to the CTL proteolytic site, and ATP depletion (Fig. 5A, arrows; and Fig. S1, left), verifying ATP dependence typical of the 26S proteasome. A smaller peak of epoxomycin-sensitive activity eluted in the apparent molecular weight of the 20S proteasome (Fig. 5A, GF 25–27, 670 kDa). This pool displayed weak ATP dependence (Fig. S1, compare GF 25–27 with 20–22), likely reflecting contamination by a small amount of poorly separated 26S proteasome. Interestingly, this activity was not stimulated by 0.02% SDS (Fig. S1, GF 25–27, + SDS), a treatment commonly used to detect the 19S-free 20S core. The basis of this resistance to activation by SDS is unknown, but similar resistance characterized WT and PiZ samples, suggesting a mechanism unrelated to AT-Z. Finally, CTL assays revealed similar levels of epoxomycin-insensitive activities in the range of molecular weights that was too small to represent the proteasome (Fig. 5A, GF 30–35).

Western blot analysis of WT and PiZ extracts separated on Superose 6 verified co-elution of the 20S β5 and alpha subunits in two major pools (Fig. 5B, 20S β5 and alphas) consistent with the peptidase activity profiles described above. In each extract, only the larger complex co-eluted with the Rpt4 subunit representative of the 19S activator (Fig. 5B, 19S Rpt4). The Rpt4 elution was broad, consistent with its incorporation in multiple large complexes, such as doubly and singly capped 26S proteasomes, and free 19S. In addition, WT and PiZ extracts contained similar amounts of unassembled Rpt4 (Fig. 5B, 19S Rpt4, GF 31–33). To quantitate the individual pools, gel filtration fractions 20–22, 25–27, and 30–33 corresponding to the 26S, 20S, and unassembled subunits, respectively, were combined and analyzed side-by-side. Similar levels of subunits were observed in WT and PiZ pools, with about 1/3 and 2/3 of the total β5 and alpha subunits present in the 26S and 20S complexes, respectively (Fig. 5C, lanes 1,2 and 3, 4), and with Rpt4 similarly distributed between the 26S complexes and free subunits (Fig. 5C, lanes 1,2 and 5,6). Thus, WT and PiZ extracts contained similar pools of the 26S, 20S, 19S, and free subunits.

In contrast to these similarities, we also observed consistent differences in WT and PiZ samples. For example, WT and PiZ extracts pre-incubated for 1 hour at 37°C with 200 mM KCl, which resulted in loss of the main peak of activity due to disassembly of the 26S complex into the 20S and 19S (Fig. 5D and E), had slightly different elution profiles of the 19S (Fig. 5E, 19S Rpt4, GF 16, 19–25). This finding suggests that the 19S complexes were altered in PiZ samples and that these alterations were easier to detect in analysis of freshly disassembled 26S complexes. We also observed differences in Western blot patterns detected with monoclonal antibodies specific to prosbox I motif. A version of this motif is present in six alpha subunits, which in SDS-PAGE typically co-migrated as three major species. These species were recognized with similar sensitivities in the WT and PiZ 20S cores (Fig. 5B, WT and PiZ, GF 25–27), but had different patterns in the 26S complexes, where either the top or bottom species dominated the samples (Fig. 5B, WT and PiZ, GF 20–22). The basis of these differences is unknown, but they were observed mainly in fresh samples, as if they reflected unstable modifications that altered Western blot detection. Overall, we observed similar, albeit not identical, pools of the 26S, 20S, 19S, and unassembled subunits in WT and PiZ mouse liver extracts.

### Reduction-sensitive modifications typical of older WT mice accumulate prematurely on selected proteasomal subunits in the livers of PiZ mice

Since PiZ mouse liver undergoes oxidative stress, reduction-sensitive modifications could have contributed to the differences in Western blot detection of selected proteasomal subunits in freshly prepared extracts. To address this possibility, we analyzed WT and PiZ mouse liver extracts separated by SDS-PAGE without, or with, sample reduction with βME.

**Figure 6:**
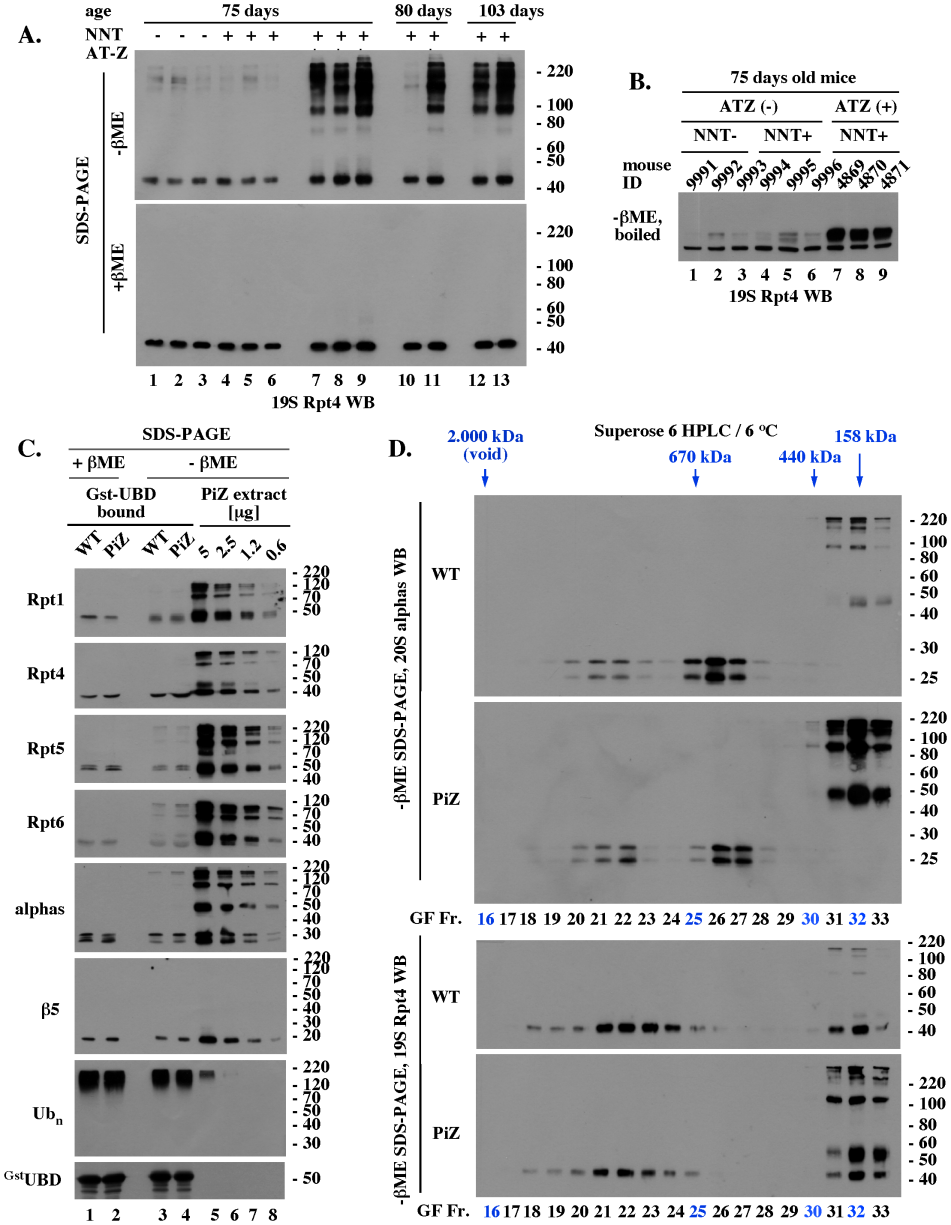
Reduction-sensitive modifications typical of aging WT mice accumulate prematurely on selected proteasomal subunits in the livers of PiZ mice. (A). Rpt4 Western blot analysis of unreduced, un-boiled liver extracts. 5 µg of the indicated extracts were mixed with Laemmli buffer without (unreduced samples) or with (reduced samples) βME (−/+ βME), separated by SDS-PAGE without prior boiling, and analyzed by Western blot with antibodies specific to Rpt4. (B). Rpt4 Western blot analysis of unreduced, but boiled, samples. Experiment like in A, lanes 1–9, except that extracts were mixed with Laemmli buffer without βME (- βME) and boiled for 4 minutes prior to SDS-PAGE. (C). Analysis of 26S proteasomes co-purified with polyubiquitin conjugates. WT and PiZ 26S proteasomes were co-purified with polyubiquitin conjugates as described in Fig. 4B and analyzed by Western blot after separation by SDS-PAGE with (lanes 1, 2) and without (lanes 3, 4) prior reduction by βME. Serial dilutions of unreduced liver extract from 103 old PiZ mouse are shown as reference (lanes 5–8). Extract prepared from 103 days old WT mice had similar reduction-sensitive modifications (data not shown, see panel A). (D). HPLC of WT and PiZ liver extracts followed by unreduced SDS-PAGE/Western blot analysis. Experiment like Fig. 5B, except that gel filtration fractions were not reduced with βME before SDS-PAGE. Data shown in A-D are representative of at least 3 independent experiments.

In the group of 75 days old mice, only livers expressing human AT-Z accumulated high molecular weight forms of Rpt4 that were detectable in unreduced samples, but not after sample reduction (Fig. 6A, Rpt4, compare lanes 7–9, and 1–6). Genetic inactivation of mitochondrial nicotinamide nucleotide transhydrogenase (NNT) involved in free radical detoxification also did not evoke accumulation of reduction sensitive changes in the absence of AT-Z (Fig. 6A, compare lanes 1–3 and 4–6). The abnormal forms of Rpt4 had different appearances in un-boiled and boiled samples, with diverse high molecular weight species detected without boiling (Fig. 6A, lanes 7–8, -βME) and smaller molecular weight alterations detected after boiling (Fig. 6B, -βME). Thus, the reduction-sensitive alterations represented a mixture of modifications that involved small and large molecular weight changes. Analysis of 80 days old mice also revealed more reduction-sensitive Rpt4 forms in the livers expressing AT-Z (Fig. 6A, lanes 10, 11). However, 103 days old mice accumulated abnormal Rpt4 forms even in the absence of AT-Z (Fig. 6A, lanes 12, 13). Thus, reduction-sensitive forms of the proteasomal Rpt4 subunit accumulated in the livers of older adult WT mice, and their accumulation was accelerated in mice transgenic with AT-Z.

In analysis of 103 days old mice, reduction-sensitive variants were observed on several subunits (Fig. 6C, lanes 5–8, Rpt1, Rpt4, Rpt5, Rpt6, and alphas), but not on β5 (Fig. 6C, lanes 5–8, β5), suggesting that not all subunits were susceptible to reduction-sensitive alterations. In the case of subunits that were altered, normal and abnormal variants were detectable with similar sensitivities in serially diluted extracts (Fig. 6C, Rpt1-6 and alphas, lanes 5–8). In contrast, normal forms of Rpt1, Rpt4, Rpt5, and alphas were enriched in unreduced 26S complexes co-purified with polyubiquitin conjugates, resembling the appearance of preparations exposed to reduction prior to SDS-PAGE (Fig. 6C, compare lanes 3, 4 with 7, 8). To verify this finding, WT and PiZ liver extracts prepared from 80 days old mice were separated by gel filtration chromatography and the collected fractions were analyzed by SDS-PAGE/Western blot without prior reduction by βME. Abnormal modifications were detected mainly on unassembled WT and PiZ subunits, with enrichment in PiZ samples (Fig. 6D, 19S Rpt4, 20S alphas, GF 31–33). The most noticeable exception was the Rpt6 ATPase, which was altered even in the 26S proteasome co-purified with polyubiquitin conjugates (Fig. 6C, Rpt6, lanes 1,2 and 3,4), where the modifications could interfere with the 26S proteasome function.

## Discussion

In this study, we sought to determine how the conditions of liver injury in PiZ mouse affect the 26S proteasome. We addressed this question using a novel extraction strategy that preserves polyubiquitin conjugates in the presence of catalytically active 26S proteasomes and separates them from deposits of insoluble AT-Z. Our analysis supports the view that protein aggregation induces a cell-wide accumulation of polyubiquitin conjugates by an indirect mechanism, and sheds light on two specific aspects of this still enigmatic phenomenon.

The first major observation relates to the long debated question of whether a defect at the 26S proteasome contributes to the accumulation of polyubiquitin conjugates? We found that polyubiquitin conjugates isolated from PiZ mouse liver had normal affinity to recombinant ubiquitin-binding domain of Psmd4, the classic polyubiquitin chain-binding receptor of the 26S proteasome, and co-purified with normal amounts of endogenous 26S proteasomes that had active proteolytic sites. Thus, polyubiquitin conjugates were accumulating 3–4 fold despite normal recruitment to catalytically active 26S proteasomes, suggesting that the 26S proteasomes were available in excess. This finding agrees with the observation that in HEK293 cells exposed to pharmacological inhibitors of the proteasome, loss of about 80% of the total activity was tolerated without an accumulation of polyubiquitinated GFP^u^ reporter, suggesting that under normal conditions about 80% of the total 26S proteasomes was idle [48]. The question is whether the accumulation of polyubiquitin conjugates resulted from a defect in some aspect of the 26S proteasome function other than binding to polyubiquitin chain and peptidase activity, or from changes in the ubiquitin-proteasome system unrelated to the 26S proteasome? For example, the process of polyubiquitination could have been up-regulated, which would be consistent with the accumulation of monomeric ubiquitin observed in PiZ liver. However, unless the 26S proteasome function was rate-liming, up-regulation of polyubiquitination shouldn't lead to an accumulation of polyubiquitin conjugates because there was sufficient number of 26S proteasomes to conquer the increased polyubiquitin load. Defective disassembly of polyubiquitin chains could cause their accumulation, but this mechanism would lead to depletion of monomeric ubiquitin, not its accumulation, arguing against this possibility. Thus, in the light of the available evidence, our data link the accumulation of polyubiquitin conjugates to a defect at the 26S proteasome.

Our study also suggests a potentially important insight into the mechanism of the 26S proteasome impairment by linking it to an accumulation of reduction-sensitive alterations on selected proteasomal subunits. The alterations represented a mixture of changes, ranging from small molecular weight modifications to high molecular weight alterations that could represent crosslinking to other proteins. Similar alterations were observed in older WT mice, implying that the alterations reflect an aspect of oxidative stress that is shared by protein aggregation-related diseases and aging. The idea that oxidative stress affects the 26S proteasome is not new [67], but we observed an accumulation of reduction-sensitive modifications in 103-days old WT mice that wouldn't be ordinarily viewed as aged. A likely explanation is that the rapid extraction strategy used in our study preserved even the least stable alterations that could be detected with antibodies specific to proteasomal subunits, but not necessarily by other, less sensitive approaches. In this view, the alterations of proteasomal subunits in adult WT mice could represent sensitive markers of early red-ox changes that precede the induction of a classic oxidative stress response.

What evidence links the reduction-sensitive changes on proteasomal subunits to a defect in the 26S proteasome function? While most of the alterations were found on unassembled alpha and ATPase subunits, at least some of these subunits were altered within the 26S complexes. The most noticeable example was the Rpt6 ATPase, which was profoundly altered even in the 26S proteasome co-purified with polyubiquitin conjugates. Why did the 26S proteasome tolerate major alterations on Rpt6, but not on the other five ATPases? A possible answer is suggested by recent high-resolution, cryo-EM and modeling studies, which revealed that Rpt6 is unique in that it adapts two very different conformations [68]–[73]. In idle 26S complexes, Rpt6 is mostly excluded from the ATPase ring. In another conformation, which is adapted upon substrate recruitment, Rpt6 becomes re-inserted into the ATPase ring, where it coordinates a series of conformational transitions necessary for substrate de-ubiquitination, unfolding and translocation to the proteolytic core. The two very different conformations could explain why major modifications on Rpt6 would have no effect on complex assembly, but yet could interfere with function. Consistent with this model, the S6 ATPase of the 26S proteasome was identified as the main intracellular target of carbonylation mediated by prostaglandin D2 metabolite, a potent inducer of oxidative stress, and shown to reduce the total ATPase activity associated with the 26S proteasome, and to induce a build-up of polyubiquitin conjugates [74]. The 19S activator has been proposed to represent the main target for oxidative stress-mediated alterations based on the finding that the 26S proteasome is more sensitive than the 20S proteasome to inhibition by H_2_O_2_ treatment [75]. Interestingly, inhibition of the 26S proteasome reported in that study was not associated with disassembly of the 26S proteasome or activation of the 20S core, in agreement with our results. Oxidative stress has been also shown to inhibit the p97/Cdc48 AAA ATPase by promoting modification of a conserved cysteine residue [76]. Since proteasomal ATPases belong to the same class, they could be regulated by a similar mechanism. It is possible that oxidative stress-mediated changes in AAA ATPases represent an early step in the mechanism by which an accumulation of protein aggregates indirectly obstructs degradation of polyubiquitinated proteins without an apparent change in the levels, assembly, and peptidase activity of the 26S proteasome. However, when oxidative damage intensifies, an eventual disassembly of the 26S complex and activation of the 20S proteasome by a mechanism independent of the 19S could be necessary for survival, as previously proposed [77].

The reduction-sensitive changes were detectable mainly on free subunits, as if oxidative stress-mediated damage interfered with some aspect of complex assembly or promoted complex disassembly. Nevertheless, we found no change in the total levels of free subunits, or the 26S and 20S proteasomes. This conclusion is based on quantitative Western blot analysis of crude extracts, affinity purified preparations, and samples separated by gel filtration chromatography. The analysis was performed with several antibodies, which limits the possibility that detection was compromised by epitope alterations. We also visualized the complexes by electron microscopy, which allowed quantitation of the single and doubly capped 26S complexes. Thus, it appears that WT and PiZ samples indeed contained similar proteasome pools. The key question is whether this state reflected a lack of response to an increased demand for the 26S proteasome function, or a response counteracted by rapid elimination of damaged subunits and/or complexes? The problem with addressing this question comes from the difficulty of testing protein turnover on a fraction of the total amount, especially in an organ as heterogeneous as the liver, which includes cells at different stages of growth, division, differentiation, and AT-Z accumulation. Nevertheless, the idea of increased proteasomal turnover is consistent with the observation that PiZ liver has an elevated autophagy, a process well suited to remove large protein complexes. If this prediction were correct, then an additional activation of autophagy by pharmacological agents, which reduces AT-Z polymer load and liver injury in the PiZ mouse [30], [31], would also increase the proteasome ‘load to capacity’ balance by reducing the 26S proteasome levels. Since the proteasome ‘load versus capacity’ balance determines apoptotic sensitivity of multiple myeloma cells in a manner linked to robust protein secretion [78]–[80], it cannot be excluded that a similar phenomenon plays a role in the PiZ liver, but the increased proteasome load there was tolerated because of a high capacity. The key question is whether the proteasome ‘load versus capacity’ balance is diminished in cells derived from homozygous ZZ individuals with the acute version of liver disease that has been linked to slower proteasomal proteolysis of AT-Z [11]. If the proteasomal ‘load to capacity balance’ were diminished, many proteasomal substrates would be degraded with slower rates, reflecting a cell-wide impairment of the 26S proteasome function rather than a defect specific to AT-Z turnover. In such a case, the proteasome increased ‘load to capacity balance’ could be the main source of toxicity, not the AT-Z accumulation, and pharmacological activation of autophagy would not compensate for this problem. It will be interesting to see these possibilities addressed in future studies.

## Supporting Information

Figure S1 **ATP-dependence of CTL activities.** The gel filtration fractions 20–22 and 25–27 were combined like in Figure 5C and analyzed for CTL activity as described in Methods. After determining the initial rates (5 minutes), mixtures were supplemented by 2 units of apyrase (marked with arrow) and monitored until the new rates of AMC accumulation stabilized (80 minutes). Reactions were then supplemented with 0.02% of SDS (marked with arrow) and monitored for additional 20 minutes (presented as 85–105 min).
(TIF)
