# Supplementary figures and images for: PiZ Mouse Liver Accumulates Polyubiquitin Conjugates That Associate with Catalytically Active 26S Proteasomes
Source: PLoS One. 2014 Sep 11;9(9):e106371. doi: 10.1371/journal.pone.0106371 (PMC4161314; doi:10.1371/journal.pone.0106371)

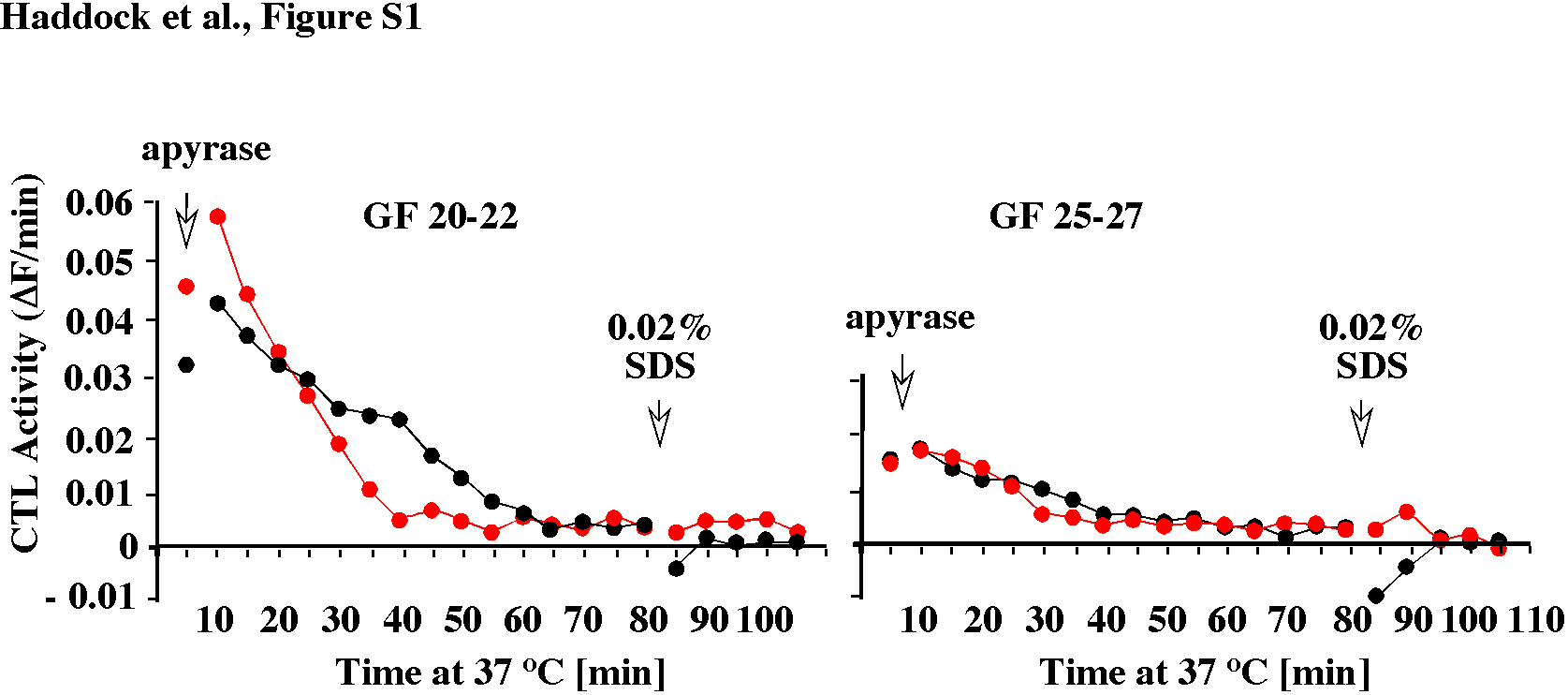

Supplement: Figure S1 — ATP-dependence of CTL activities. The gel filtration fractions 20–22 and 25–27 were combined like in Figure 5C and analyzed for CTL activity as described in Methods. After determining the initial rates (5 minutes), mixtures were supplemented by 2 units of apyrase (marked with arrow) and monitored until the new rates of AMC accumulation stabilized (80 minutes). Reactions were then supplemented with 0.02% of SDS (marked with arrow) and monitored for additional 20 minutes (presented as 85–105 min). (TIF) [file pone.0106371.s001.tif]
